# SARS-CoV-2 Positivity in Early Infancy: A National Cohort From Saudi Arabia

**DOI:** 10.3389/fped.2022.849659

**Published:** 2022-03-28

**Authors:** Lana A. Shaiba, Adnan Hadid, Khalid Altirkawi, Mahdi A. Alnamnakani, Abdulaziz A. Almutayliq, Areen T. Alharbi, Asmar M. Hijazi, Khalid M. AlMoosa, Nora F. AlSaud, Rozan E. Murshid, Wejdan S. AlMuhanna, Nasser A. Aldawsari, Maryam F. Bin Hadyan, Rana Almaghrabi, Yousef M. Alsofayan, Ahmed A. Alahmari, Yasir S. Almuzaini, Fahad A. Alamri, Anas A. Khan, Prakesh S. Shah

**Affiliations:** ^1^Department of Pediatrics, College of Medicine, King Saud University, Riyadh, Saudi Arabia; ^2^Department of Neonatology, King Saud University Medical City, Riyadh, Saudi Arabia; ^3^Department of Pediatrics, King Saud University Medical City, Riyadh, Saudi Arabia; ^4^Department of Pediatrics, King Fahad Medical City, Riyadh, Saudi Arabia; ^5^Department of Pediatrics, Pediatric Infectious Disease, Prince Sultan Military Medical City, Riyadh, Saudi Arabia; ^6^Global Center for Mass Gatherings Medicine, Ministry of Health, Riyadh, Saudi Arabia; ^7^Department of Emergency Medicine, College of Medicine, King Saud University, Riyadh, Saudi Arabia; ^8^Department of Pediatrics, Mount Sinai Hospital, Toronto, ON, Canada; ^9^Department of Pediatrics, Toronto University, Toronto, ON, Canada; ^10^Maternal-Infant Care Research Centre, Mount Sinai Hospital, Toronto, ON, Canada

**Keywords:** SARS-CoV-2, positivity, early infancy, national, cohort, Saudi Arabia

## Abstract

**Background:**

Data on SARS-CoV-2 in infants ≤ 90 days are limited with conflicting reports regarding its presentation and outcomes.

**Methods:**

We conducted an ambispective cohort study using prospectively collected Health Electronic Surveillance Network Database by the Ministry of Health, Saudi Arabia. Infants of ≤ 90 days of age who had a positive RT-PCR test for SARS-CoV-2 virus were included. Patients were divided in Early neonatal (0–6 days), late neonatal (7–27 days), and post- neonatal (28–90 days) groups and were compared for clinical characteristics and outcomes by contacting parents and collecting information retrospectively.

**Results:**

Of 1,793 infants, 898 infants were included for analysis. Most infants in the early neonatal group had no features of infection (tested based on maternal positivity), whereas most infants in the late and post- neonatal groups were tested because of clinical features of infection. Fever and respiratory signs were the most common presenting feature in the late and post-neonatal groups. Hospitalization was higher in the early neonatal group (80%), compared to the two other groups. The overall mortality in the cohort was 1.6%.

**Conclusion:**

SARS-CoV-2 infection in infants ≤ 90 days might not be as rare as previously reported. The clinical presentation varies based on age at positive RT-PCR result.

## Introduction

The first reported pediatric case of coronavirus diseases of 2019 (COVID-19) was a 10-year-old boy from Shenzhen, China, diagnosed with the condition in January 2020 (1). Available literature suggests that pediatric population may be less affected from COVID-19 than adult population, (2) and that a high percentage of infected children remain asymptomatic. The majority of children develop a milder disease and have a lower mortality rate (3, 4). However, the newly mutated variants seem to affect children more than what has been reported earlier (5).

Approximately a third of hospitalized children due to COVID-19 were ≤ 90 days of age (6) and half of the patients in this age group and in neonates when presented to hospital required admission (7, 8). Infants ≤ 90 days of age are at a higher risk of developing severe COVID-19 disease (9–11). Furthermore, the reported mortality rate of infants in this age group has ranged between 0 and 2.9% (6, 12, 13). However, data specifically reporting on infants of ≤ 90 days of age have been limited (14).

The objective of this study is to describe the clinical features, presentation, and outcomes of infants of ≤ 90 days of age who were identified to be severe acute respiratory syndrome coronavirus 2 (SARS-CoV-2) positive from a national cohort from Saudi Arabia.

## Methods

### Design, Setting, and Inclusion Criteria

We conducted an ambispective cohort study from a prospectively collected database by the Ministry of Health in Saudi Arabia. We also obtained detailed information from families via phone interviews if we identified missing information. The study included infants of ≤ 90 days of age who had a positive SARS-CoV-2 reverse transcriptase polymerase chain reaction (RT-PCR) test results and presented to health care facilities between March 12, 2020, and February 8, 2021, in Saudi Arabia. No exclusion criteria were applied. We collected data only on the first infection, as re-infection in this age group has not been reported.

### Procedure

After obtaining the required permits and ethics committees' approval, data were extracted from the Health Electronic Surveillance Network (HESN) Database housed within the Ministry of Health in Saudi Arabia. The database contains demographic, clinical features, and outcome data of patients who were tested positive for SARS-CoV-2 via RT-PCR testing. The data were entered in the system by trained health care workers from all regions of Saudi Arabia (15). We extracted infants from the database with positive RT-PCR for SARS-CoV-2 and then called those who were ≤ 90 days of age into a datasheet. From this parent database, data on infants of ≤ 90 days of age were extracted by two abstractors involved in this study in separate electronic datasheets. These abstracted data were checked by a third investigator to ensure completeness and to remove duplicate records. During the study period, the positivity of SARS-CoV-2 was assessed using RT-PCR test from nasopharyngeal samples in accordance with protocols set by the World Health Organization throughout Saudi Arabia (16). Using a standardized questionnaire, one of the investigators or research associates made a phone call to parents/caregivers of all infants with positive results. The phone calls were made between 22/02/2021 and 29/04/2021 to complement information obtained from HESN. The additional information included: demographics, gestational age, mother's COVID-19 status at the time of birth, feeding method at time of testing, contact with any other COVID-19 patients, clinical presentation, hospital/ICU admission, and mortality. Before labeling a phone number as “no response” the phone number was tried four times on different occasions. The HESN data items were also checked with infants' caregivers to remove duplicate information if an infant attended multiple facilities. The data collected from parents were inputted on a google sheet form which was then exported to an excel sheet. The data sheet was reviewed daily by two of the investigators to ensure completeness of the data collected.

### Outcomes and Groups

Infants in the study were divided into early neonatal (0–6 days), late neonatal (7–27 days), and post-neonatal (28–90 days) groups, according to their age at first positive RT-PCR test for SARS-CoV-2 as per the AAP definition (14). The status on hospitalization was collected as hospitalized or not hospitalized.

### Ethics

The study was approved by the Central Ministry of Health Institutional Review Board Committee (Approval number 20−198M), Saudi Arabia. During the phone interview, a verbal consent was obtained first from the parents/caregivers and if parents declined participation, the patient was excluded from the study. Throughout the study, data were stored on a computer with a strong password and access was restricted to authorized investigators after signing non-disclosure agreement forms.

### Statistical Analysis

Descriptive statistics were performed at first. Three groups, based on timing of testing of positivity, were compared against each other. We also evaluated the outcomes of three groups based on illness severity (asymptomatic, symptomatic, hospitalized, and non-hospitalized). Finally, we compared outcomes based on the status of hospitalization; hospitalized vs. not hospitalized. We reported continuous variable outcomes as mean and SD (for normally distributed data) or median and IQR (for non- normally distributed data), and categorical variable outcomes as percentages. We used Student *t* test, chi-square test or Wilcoxon Rank Sum test as appropriate for these comparisons. *P* < 0.05 were considered significant. All analyses were conducted using SPSS software (SPSS Statistics for Windows, Version 26.0. Armonk, NY).

## Results

**Figure 1:**
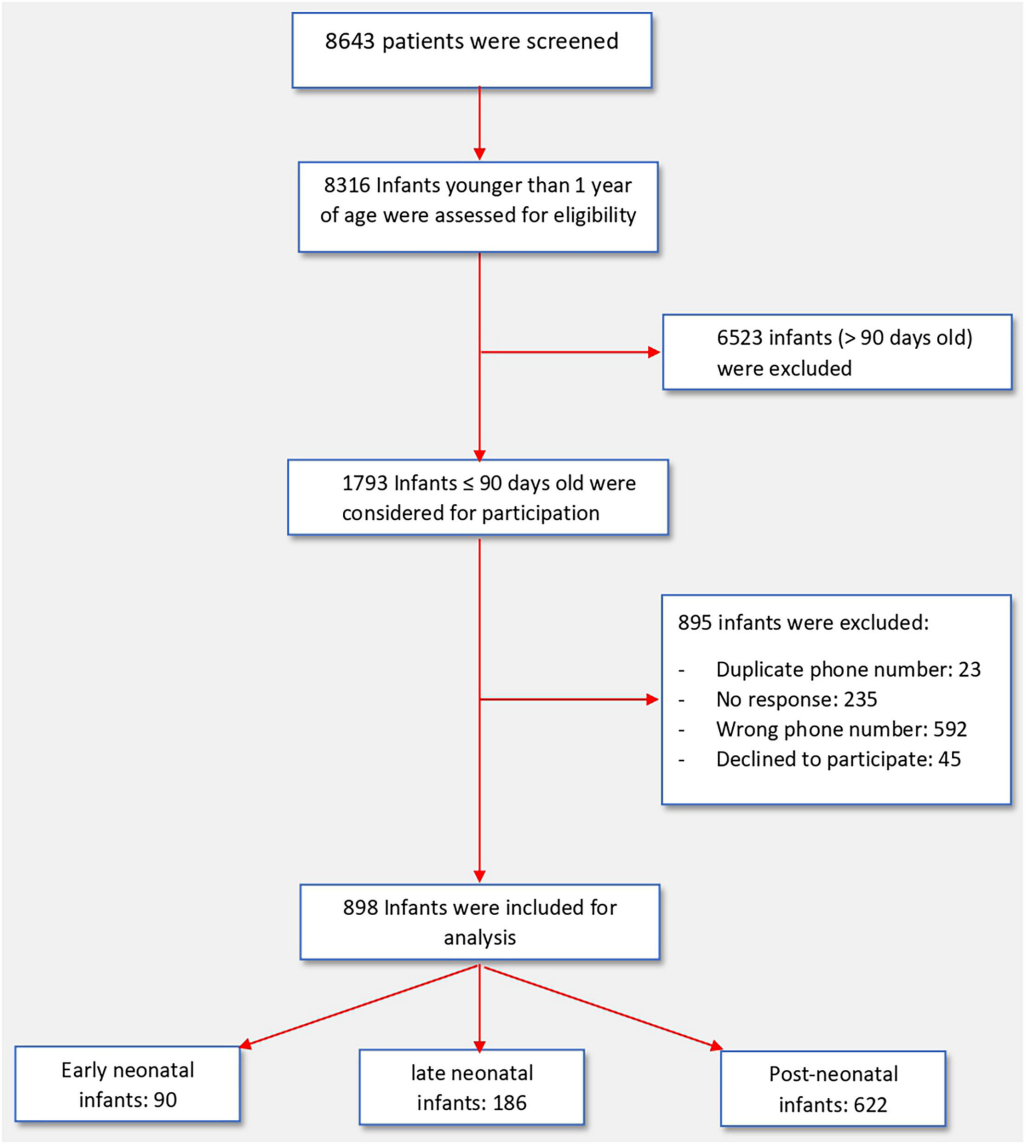
Flow chart showing patient recruitment.

On the initial scan of the HESN database, a total of 1,793 infants of ≤ 90 days of age were identified, of them, 895 infants were excluded due to duplicate records, incorrect contact details, no response or refusal to participate. Thus, we included 898 infants in this study (Figure 1). The baseline characteristics of all three groups of infants are presented in Table 1. Included were 90 patients in the early neonatal group, 186 patients in the late neonatal group and 622 patients in the post neonatal group. Early neonatal group patients were mostly tested due to maternal positivity, whereas late neonatal and post-neonatal patients were tested either because of symptoms or being in contact with a person with positive RT-PCR test. Fever and respiratory signs were the most common presenting features in late neonatal and post-neonatal groups; however, nearly one third of them presented with irritability or gastrointestinal signs.

**Table 1:** Characteristics of study population.

**Characteristics**	**Early neonatal <7 days**	**Late neonatal 7–27 days**	**Post neonatal 28–90 days**	***P***
	***N* = 90**	***N* = 186**	***N* = 622**	
General characteristics				
Age at time of testing, days; median (IQR)	1 (1–3)	18 (12–21)	60 (37–69.25)	–
GA at birth, weeks; mean (SD)	38.4 (2.5)	39.0 (2.4)	38.3 (2.4)	0.026
Preterm gestation, *n* (%)	16 (17.8%)	11 (5.9%)	60 (9.6%)	0.002
Male sex, *n* (%)	41 (45.6%)	94 (50.5%)	357 (57.4%)	0.085
Maternal COVID-19 positive at birth, *n* (%)	55 (61.1%)	2 (1%)	10 (1.6%)	<0.001
Asymptomatic, *n* (%)	52 (57.8%)	27 (14.5%)	61 (9.8%)	<0.001
Reason for testing				
Contact, *n* (%)	11 (21.1%)	39 (21%)	194 (31.2%)	<0.001
Symptomatic, *n* (%)	22 (24.0%)	133 (71.5%)	393 (63.2%)	
Suspicion, *n* (%)	2 (2.4%)	8 (4.3%)	11 (1.8%)	
Other, *n* (%)	47 (52.2%)	6 (3.2%)	24 (3.9%)	
Exposure to family members with active COVID-19, *n* (%)	51 (56.7%)	145 (78%)	507 (81.5%)	<0.001
Any comorbidity, *n* (%)	3 (3.3%)	16 (8.6%)	54 (8.7%)	0.247
Clinical features^*^				
Asymptomatic, *n* (%)	52 (57.8 %)	27 (14.5%)	61(9.8%)	<0.001
Fever, *n* (%)	27 (30%)	125 (67.2%)	451 (72.5%)	<0.001
Respiratory signs, n (%)	22 (24.4%)	93 (50%)	326 (52.4%)	0.341
Runny nose, *n* (%)	11 (12.2%)	47 (25.3%)	161 (25.9%)	
Cough, *n* (%)	5 (5.6%)	46 (24.7%)	165 (26.5%)	
Tachypnea, *n* (%)	15 (16.7%)	58 (31.2%)	145 (23.3%)	
Nasal congestion, *n* (%)	9 (10%)	28 (15%)	120 (19.3%)	
Sneezing, *n* (%)	0 (0%)	3 (1.6%)	6 (1%)	
Cyanosis, *n* (%)	0 (0%)	2 (1%)	7 (1.1%)	
Irritability, *n* (%)	8 (8.8%)	57 (30.6%)	208 (33.4%)	<0.001
Gastrointestinal symptoms, *n* (%)	12 (13.3%)	54 (29%)	196 (31.5%)	0.002
Diarrhea, *n* (%)	12 (13.3%)	41 (22%)	164 (26.4%)	
Decreased feeding, *n* (%)	2 (2.2%)	23 (12.4%)	133 (21.4%)	
Vomiting, *n* (%)	2 (2.2%)	24 (12.9%)	77 (12.4%)	
Lethargy, *n* (%)	7 (7.8%)	35 (18.8%)	113 (18.2%)	0.036
Skin Rash, *n* (%)	1 (1.1%)	16 (8.6%)	46 (7.4%)	0.056
Conjunctivitis, *n* (%)	0 (0%)	11 (5.9%)	47 (7.6%)	0.028
Seizures, *n* (%)	0 (0%)	0 (0%)	2 (0.3%)	>0.999

* *Many patients had multiple clinical features*.

As seen in Table 2, there were 140 asymptomatic infants and 758 symptomatic infants. About two fifths of infants in the early neonatal group had features suggestive of infection whereas the majority of the infants in the late neonatal and post neonatal groups had features suggestive of infection, 58, 85, and 90%, respectively. The percentage of infants with clinical features who were born at preterm gestation was higher in the post-neonatal group (10%) compared to both early and late neonatal groups (7.9 and 3.8% respectively). Exclusive formula feeding was higher in early neonatal period (78.8%) (Table 2).

**Table 2:** Asymptomatic and symptomatic patients grouped according to postnatal age.

	**Asymptomatic**			**Symptomatic**		
	***N*** **= 140**			***N*** **= 758**		
	**Early neonatal <7 days**	**Late neonatal 7–27 days**,	**Post neonatal 28–90 days**	**Early neonatal <7 days**	**Late neonatal 7–27 days**	**Post neonatal 28-90 days**
	**(*N* = 22)**	**(*N* = 27)**	**(*N* = 60)**	**(*N* = 38)**	**(*N* = 159)**	**(*N* = 561)**
Age at time of testing, days; median (IQR)	1 (1)	20 (10–21)	60 (40–75)	3 (1–4)	17 (12–21)	60 (37–68)
GA at birth, weeks; mean (SD)	37.9 (2.8)	37.5 (3.8)	38.7 (2.4)	39.1 (1.7)	39.2 (2.1)	38.2 (2)
Preterm gestation, *n* (%)	13 (25%)	5 (18.5%)	4 (6.6%)	3 (7.9%)	6 (3.8%)	56 (10%)
Male sex, n (%)	25 (48%)	15 (55.6%)	40 (65.6%)	16 (42.1%)	79 (49.7%)	317 (55.5%)
Maternal COVID-19 at birth, *n* (%)	39 (75%)	0 (0%)	3 (4.9%)	16 (42.1%)	2 (1.3%)	7 (1.2%)
Exposure of family members with active COVID-19, *n* (%)	17 (32.7%)	01 (70.4%)	48 (78.7%)	20 (52.6%)	109 (68.6%)	446 (79.5%)
Type of feeding received^*^						
• Breast feeding exclusive, *n* (%)	5 (11.7%)	17 (63%)	24 (39.3%)	15 (39.5%)	57 (35.8%)	165 (29.4%)
• Formula feeding exclusive, *n* (%)	41 (78.8%)	5 (18.5%)	20 (32.8%)	18 (47.4%)	42 (26.4%)	174 (31%)
• Mixed feeding *n* (%)	5 (9.6%)	5 (18.5%)	17 (27.9%)	5 (13.2%)	60 (37.7%)	222 (39.6%)

* *One patient in asymptomatic early neonatal group was not fed when diagnosed*.

Of the included infants 402 were hospitalized while 496 were not hospitalized. Among the hospitalized infants, the median age at testing was 30 days. Being born at preterm gestation was more prevalent among hospitalized infants (13%) vs. (7%) in non-hospitalized infants. Infants with comorbidity, or those with a positive maternal COVID-19 swab at birth, were more likely to be hospitalized than non-hospitalized, however, this may reflect the initial birth hospitalization. More infants had a history of exposure to family member with active COVID-19 at presentation in the non-hospitalized group (82%) compared to the hospitalized group (62.9%). The characteristics of hospitalized patients are detailed in Table 3.

**Table 3:** Characteristics of population among hospitalized and non-hospitalized.

**Characteristics of population**	Hospitalized^*^	**Non-hospitalized**	***P***
	**(*N* = 402)**	**(*N* = 496)**	
Age at time of testing, days; median (IQR)	30 (11–50)	50 (30–65.75)	<0.001
GA at birth, weeks; mean (SD)	38.3 (2.8)	38.7 (2.2)	0.027
Preterm, *n* (%)	54 (13.4%)	33 (6.7%)	<0.001
Male sex, *n* (%)	221 (55%)	271 (54.6%)	0.925
Maternal COVID-19 at birth, *n* (%)	52 (12.9%)	15 (3%)	<0.001
Exposure of family members to an active COVID-19 case, *n* (%)	253 (62.9%)	406 (82%)	<0.001
Any comorbidity, *n* (%)	41 (10.2%)	23 (4.6%)	0.002
Type of feeding received			
Breast feeding exclusive, *n* (%)	126 (31.3%)	157 (31.7%)	<0.001
Formula feeding exclusive, *n* (%)	159 (39.6%)	141 (28.4%)	
Mixed feeding, *n* (%)	116 (28.9%)	198 (39.9%)	
Age of sample population			
Early neonatal <7 days, *n* (%)	72 (17.9%)	18 (3.6%)	<0.001
Late neonatal 7–27 days, *n* (%)	107 (26.6%)	79 (15.9%)	
Post neonatal 28–90 days, *n* (%)	223 (55.5%)	399 (80%)	

* *One patient in hospitalized group was not fed when diagnosed*.

As presented in Table 4. Infants of the early neonatal period were more likely (80%) to be hospitalized, as compared to those of late neonatal (57.5%), or post neonatal (35.9%) periods.

**Table 4:** Outcomes according to age at time of presentation.

**Clinical features**	**Early neonatal <7 days**	**Late neonatal 7–27 days**	**Post neonatal 28–90 days**	***P***
	***n* = 90**	***n* = 186**	***n* = 622**	
Hospitalization *n* (%)	72 (80 %)	107 (57.5%)	223 (35.9%)	<0.001
ICU hospitalization among those who were admitted, *n* (%)	24/72 (33.3%)	22/107 (20.6%)	32/235 (14.3%)	0.002
Length of stay among hospitalized, days; median (IQR)	7 (4–14)	7 (4–10)	6 (3–10)	0.714
Mortality, *n* (%)	4 (4.4%)	4 (2.2%)	6 (1%)	0.045

Furthermore, infants in the early neonatal period were more likely to be admitted to the ICU as compared with infants of the other two groups. The median length of hospital stay was not significantly different among the groups. The overall mortality of the whole cohort was 14 (1.56%) infants, with a higher rate of death among infants of the early neonatal period (Table 4).

Table 5 describes the details of the infants who died. Four infants were born to COVID-19 positive mothers and were tested positive after delivery. Most of the infants who died had respiratory distress (10/14 = 71.4%), five of them presented with fever, and two presented with GI symptoms of vomiting and diarrhea. Six of the non-survivor infants were born preterm, and four of them had a comorbidity including metabolic disorder or a congenital heart disease.

**Table 5:** Descriptive details of non-survivors.

**Case number**	**Type of delivery**	**Gender**	**Age at time of swabbing (by days)**	**Gestational age (week)**	**Feeding (when he/she got infected)**	**Underlying disease**	**Mother SARS-cov-2 status at delivery/Baby's SARS-CoV-2 status after delivery**	**Reason for testing**	**Symptoms at presentation**	**Required oxygen**	**Length of hospital stay in days**	**Length of ICU stay in days (in case of admission)**
1	SVD	F	1	40	Formula	None	Yes/Positive	Born to COVID-19 positive mother	Runny nose, nasal congestion, difficulty of breathing, irritability	Yes	1	1
2	CS	F	1	30	NA	None	Yes/Positive	Born to COVID-19 positive mother	Hypotensive and bradycardia	Yes	1	1
3	SVD	F	1	40	Exclusive breast feeding	None	Yes/Positive	Born to COVID-19 positive mother	Fever Cough, runny nose, difficulty of breathing	Yes	4	4
4	CS	F	1	33	Formula	None	Yes/Positive	Born to COVID-19 positive mother	Difficulty Breathing	Yes	14	14
5	SVD	M	14	40	Exclusive breast feeding	Congenital heart disease	NA/NA	Symptomatic/ contact	Fever Difficulty of breathing, cyanosis	Yes	18	18
6	CS	M	25	40	Formula	Congenital heart disease	NA/NA	Symptomatic/ contact	Difficulty of breathing, cyanosis	Yes	3	2
7	SVD	F	30	37	Mixed	None	NA/NA	Hospital Admission/ Transfer	Fever, PR bleed and nasal bleed	Yes	16	10
8	SVD	M	60	34	Mixed	Metabolic disease	NA/NA	Hospital Admission/ Transfer	Irritability, decreased feeding, severe anemia	Yes	18	18
9	SVD	F	35	37	Mixed	None	NA/NA	Symptomatic	Difficulty of breathing, Irritability, Hypoactive, decreased feeding, seizures	Yes	10	6
10	CS	M	41	40	Formula	Congenital heart disease	NA/NA	Symptomatic	Fever Difficulty of breathing	Yes	1	1
11	SVD	F	60	Term	Mixed	None	NA/NA	Symptomatic/ contact	Fever Diarrhea	No	2	No
12	SVD	M	90	32	Formula	None	No/NA	Symptomatic/ contact	Difficulty of breathing, cough, runny nose, nasal congestion	Yes	3	No
13	CS	M	14	30	Formula	None	No/NA	Symptomatic/ contact	Difficulty of breathing Vomiting	Yes	17	17
14	CS	F	14	30	Formula	None	No/NA	Symptomatic/ contact	Difficulty of breathing	Yes	17	17

## Discussion

We report in this study on one of the largest nation-wide cohorts with a diverse population, which allows for a balanced epidemiological profile of SARS-CoV-2 infection in infant ≤ 90 days of age. Our study suggests that while only two fifth of early neonatal cases of COVID-19 disease were symptomatic, most infants who are < 90 days old presented with symptoms when infected with SARS-CoV-2 virus. Symptoms' frequency, however, varied among groups; in early neonatal group, the most common presenting sign was fever followed by respiratory symptoms, whereas in late and post neonatal groups respiratory symptoms were more common followed by fever.

In a case series, Shaiba et al. (17) reported that most (94%) infants of ≤ 90 days old presented to a local hospital for SARS-CoV-2 testing were symptomatic. This is almost similar to what was observed in this study where 85% of all infants presented with symptoms (42, 85, and 90% in early, late and post neonatal groups, respectively). In the aforementioned case series, the most common presenting sign was fever (70%) followed by respiratory signs (59%), which is comparable to our study's findings, where fever was encountered in 67% of infants followed by respiratory signs in 50% of them. Gastrointestinal symptoms, however, presented in only 30% of our study's population, in contrast to what has been published in a Canadian study, where GI symptoms were more prevalent (85%) (8). As for the cutaneous and neurological manifestations of COVID-19 disease in infants ≤ 90 days, our study has corroborated previous reports, which indicated that these signs and symptoms are very rare (8, 9, 13, 15).

In a recent systematic review of SARS-CoV-2 infection in neonates, they have reported that 50% of neonates were symptomatic upon presentation (18). The most prominent clinical feature in this systematic review was respiratory signs and symptoms.

As expected, most of the infants in the early neonatal group had a mother who tested positive just prior to birth, this finding suggests the possibility of maternal-fetal transmission in some if not all of these cases. This possibility of maternal-fetal transmission is further supported by the positive SARS-CoV-2 results of infants born to a mother with SARS-CoV-2 infection despite the strict and immediate isolation of the infant from the mother upon birth (as per the Saudi National regulation by the Saudi Ministry of Health). Contrary to the published literature, our study reports a higher percentage of infants tested positive with a family member who is affected by COVID-19 (8, 13, 19).

Breastfeeding and Kangaroo mother care (KMC) are integral parts of neonatal care and has been a standard of care in recent years (20, 21). Neonatologists all over the world had confusion in the initial days of the pandemic to advocate for breastfeeding and KMC as some initial guidelines early in the pandemic advised immediate mother and infant separation for neonates born to mothers infected with SARS-CoV-2. This recommendation seems unnecessary especially with current evidence suggesting only 2.16% of breastmilk samples tested for SARS-CoV-2 RT-PCR were positive and 5% of breastmilk samples contained viral genome with low infectivity rate (22, 23). In our population exclusive breastfeeding rate was low (24). It has been noted that the rate of breastfeeding has gone even lower as only 32.9% of asymptomatic mothers were exclusively breastfeeding and 31.3% of symptomatic mothers were exclusively breastfeeding. Many of neonates and infants were separated from their mothers after being diagnosed with SARS-CoV-2 which prevented proper KMC which might have contributed to the lower rate of breastfeeding. Of note, one of the neonates (neonate number 1 in Table 5) who died on day one of life although the mother expressed breastmilk the neonate was never fed due to his critical condition.

Hospitalized infants tended to be younger (11–50 days of age) than the non-hospitalized ones (30–66 days), this higher rate of admission in younger infants is similar to what was observed in previous studies. Panetta et al. reported that infants who were ≤ 90 days were more likely to be hospitalized (57%) compared to older infants (15%) (8). In a study investigating severe cases of SARS-CoV-2 infection in children 0–18 years of age, Ouldali et al. reported that infants <90 days of age accounted for 37% of admissions with SARS-CoV-2 (6). In our study, the majority of early neonatal infection cases were hospitalized (80%), and of them, one third required ICU admission. Policy aside, we speculate that these infants were admitted to the ICU due to reasons other than SARS-CoV-2 infection, such as prematurity. Of note, a recent study from Saudi Arabia derived from the same pool of patients reported on infants 2 months of age and older suggested that hospitalization rate of infants 2 month of age-−1 year is 11% (3/27 infants) (4). Of note the period of data collection in the study cited was only 1 month and reported cases during the first wave of SARS-CoV-2, when the pediatric patients were less.

COVID-19 infected infants (<1year of age) comprise 17% of all pediatric cases <18 years of age. Also, in infants <1 year of age critical cases were seen in 14% compared to 5% in children older than 1 year of age (25). The neonatal mortality rate (early and late) in our study was 2.9%, which is higher than the rate reported in a national cohort from the UK (2.4%) (9). Notably, the investigators have not specified in this study whether the mortality was directly related to COVID-19 infection (12). This finding is further supported with another national cohort study in the UK (10). In our study included were 8 neonates who died, five of them were preterm and 2 had underlying congenital heart disease. The presence of these comorbidities might have contributed to the high mortality rate in this age group. The lack of a formal detailed medical report to assess the exact cause of death in our study population makes it difficult to ascertain SARS-CoV-2 infection as the primary cause of death.

Assessing the mortality rate of infants ≤ 90 days of age due to SARS-CoV-2 infection is difficult due to the limited number of studies reporting on the disease in this age group and their outcomes. The overall mortality rate in infants ≤ 90 days of life ranged in some studies from 0% (26) up to 2.9% (6, 9) in our study population, this rate is 1.6%, which is in concordance with the above studies.

Our study has many limitations. This includes the potential of selection bias, nonetheless, we strived to include all infants included in the registry by repeated calls (up to 4 times) before labeling a number as a non- responding number. Given it is a partly questionnaire-based study, there is a possibility for recall bias, however, we tried to mitigate this possibility by standardizing the way we applied the questionnaire, and by providing multiple sessions of training to the research associates contacting the families, as well as the availability of one-to-one advice around the clock by an expert physician. Lastly, we could not collect detailed clinical, laboratory and imaging data, as the HESN database is collected primarily for administrative purposes.

In conclusion, although previously reported that neonates and infants have a favorable outcome when infected with SARS-CoV-2, healthcare providers might need to be cautious when counseling parents. We report a higher rate of severe cases of SARS-CoV-2 infection in this age group. We also report a mortality rate in this vulnerable age group that is higher than some of the previously published figures. SARS-CoV-2 continues to be a threat for infants ≤ 90 days old given their susceptibility.

## Data Availability Statement

The original contributions presented in the study are included in the article/supplementary material, further inquiries can be directed to the corresponding authors.

## Ethics Statement

The studies involving human participants were reviewed and approved by King Saud University Medical City. Written informed consent to participate in this study was provided by the participants' legal guardian/next of kin. Written informed consent was obtained from the individual(s), and minor(s)' legal guardian/next of kin, for the publication of any potentially identifiable images or data included in this article.

## Author Contributions

LS and AH elicit the main idea. KA, MA, and AAAlm wrote the proposal. AAlh, AH, KMA, NAlS, and RM collected the data. WA, NAld, MB, and RA data analysis. YA, AAla, YA, FA, AK, and PS wrote the manuscript. All authors contributed to the article and approved the submitted version.

## Conflict of Interest

The authors declare that the research was conducted in the absence of any commercial or financial relationships that could be construed as a potential conflict of interest.

## Publisher's Note

All claims expressed in this article are solely those of the authors and do not necessarily represent those of their affiliated organizations, or those of the publisher, the editors and the reviewers. Any product that may be evaluated in this article, or claim that may be made by its manufacturer, is not guaranteed or endorsed by the publisher.
